# Downscaling precipitation and temperature in the Andes: applied methods and performance—a systematic review protocol

**DOI:** 10.1186/s13750-023-00323-0

**Published:** 2023-12-12

**Authors:** Santiago Núñez Mejía, Carina Villegas-Lituma, Patricio Crespo, Mario Córdova, Ronald Gualán, Johanna Ochoa, Pablo Guzmán, Daniela Ballari, Alexis Chávez, Santiago Mendoza Paz, Patrick Willems, Ana Ochoa-Sánchez

**Affiliations:** 1 TRACES & Faculty of Science and Technology, Universidad del Azuay, Av. 24 de Mayo 7-77 y Hernán Malo, Cuenca, Ecuador; 2 Department of Water Resources and Environmental Sciences, University of Cuenca, Cuenca, Ecuador; 3 Department of Geodesy and Geoinformation, Research Unit Remote Sensing, University of Technology (TU Wien), Vienna, Austria; 4 Instituto de Estudios de Régimen Seccional del Ecuador (IERSE) &, Facultad de Ciencia y Tecnología, Universidad del Azuay, Cuenca, 010101 Ecuador; 5 Hydraulics and Geotechnics Section, Department of Civil Engineering, KU Leuven, 3001 Leuven, Belgium

**Keywords:** Scale reduction, Climate change, Climate models, Reanalysis, Spatial resolution, South America, Mountain, Rain, Temperature

## Abstract

**Background:**

Global warming and climate change are threats to the world. Warmer temperatures and changes in precipitation patterns alter water availability and increase the occurrence of extreme weather events. South America and the Andes are vulnerable regions to climate change due to inequity and the uneven distribution of resources. Climate change evaluation often relies on the use of general circulation models (GCMs). However, the spatial resolution is too coarse and does not provide a realistic climate representation at a local level. This is of particular importance in mountain areas such as the Andes range, where the heterogeneous topography requires a finer spatial resolution to represent the local physical processes. To this end, statistical and/or dynamical downscaling methods are required. Several approaches and applications of downscaling procedures have been carried out in the countries of this region, with different purposes and performances. However, the main objective is to improve the representation of meteorological variables such as precipitation and temperature. A systematic review of these downscaling applications will identify the performance of the methods applied in the Andes region for the downscaling of precipitation and temperature. In addition, the meta-analysis could detect factors influencing the performance. The overall goal is to highlight promising methods in terms of fitness for use and identify knowledge gaps in the region.

**Methods:**

The review will search and examine published and grey literature on downscaling applications of temperature and precipitation in the Andes region. Predetermined criteria for eligibility will allow the screening of the evidence. Then, the method used in each application will be coded and mapped according to the country, purpose, variable, and type of downscaling. At the same time, quantitative and qualitative data will be extracted. The performance metrics are particularly interesting for this review. A meta-analysis will be conducted for those studies with comparable metrics. A narrative synthesis, maps and heatmaps will show the results. Tables, funnel plots, and meta-regressions will present the meta-analysis. Throughout the review, a critical appraisal step will categorize the validity of the evidence.

**Supplementary Information:**

The online version contains supplementary material available at 10.1186/s13750-023-00323-0.

## Background

Climate change is defined as the continuous variation in the state and properties of the climate over long periods, such as decades or longer, associated with increasing greenhouse gas emissions combined with anthropogenic activities [1]. Climate change threats can exacerbate global vulnerability, particularly in high-latitude, high-altitude, or near-sea-level locations [2, 3]. Two climate variables: precipitation and temperature, are fundamental for addressing future climate risks on the environment and natural resources [4], since their change in magnitude and distribution cause cascading impacts in natural and human systems.

According to the Intergovernmental Panel on Climate Change [5], the global surface temperature will increase under all emissions scenarios, and it will exceed the warming threshold of 1.5 °C and 2.0 °C relative to the 1850–1900 period in the twenty-first century unless strong mitigation occurs. The scenarios with the higher radiative forcing (SSP5-8.5 and SSP3-7.0) project an exceedance of the 2.0ºC global warming in the mid-term (2041–2060). Similarly, projections and analyses of precipitation suggest a rise in intensity and modifications in the spatial and seasonal distribution with certain regional variations [6–8]. As a result, extreme weather events such as floods, droughts, windstorms, and wildfires have compromised the availability of natural resources and adaptive capacity [9–11].

South America and the Andes region are vulnerable to climate change due to the uneven distribution of water resources and socioeconomic factors such as poverty, inequity, and limited access to essential services [12]. Water security achievement is among the key risks for the region [13] and this is magnified in the Andes where many big cities such as Antofagasta, Bogotá, Cuenca, La Paz, Lima, Mendoza, Quito, and others that rely mainly on the water supply from the mountains. A change in the precipitation patterns and magnitude will affect the water availability and the occurrence of extreme events in the Andean catchments.

Climate change studies rely on models for climate projections and reanalysis datasets to complement observations. General circulation models (GCMs) and regional climate models (RCMs) are three-dimensional dynamical representations of physical processes simulating the global climate system [14]. GCMs capture large-scale patterns of the sea, ice, terrestrial systems and global and continental temperature responses to changes in greenhouse gas emissions [15]. The performance of a model depends on how successfully it represents the relevant large-scale and mesoscale processes for a certain region.

In the tropical Andes, climate is mainly controlled by the Inter-tropical convergence zone (ITCZ), the Hadley cell, the El Niño Southern Oscillation (ENSO), the Atlantic Meridional Oscillation (AMO), the South American Monsoon System, the South American, Caribbean and Orinoco Low-Level Jets, and the Pacific Decadal Oscillation (PDO) [16]. Whereas in the mid-latitudes, climate is mainly influenced by moisture transport by the westerly winds from the southern Pacific Ocean. Climate models have problems representing tropical processes and therefore the magnitude and variability of precipitation and temperature in the Andes [17, 18]. As GCMs are the boundary conditions for downscaling methods, the representation of precipitation and temperature might be especially uncertain for the region. In addition, the resolution is too coarse to represent local processes in mountainous terrain and this represents complexity for decision-makers [19], thus demanding a finer spatial resolution.

Downscaling approaches are required to overcome these difficulties. Spatial downscaling uses finer-resolution data for analyzing features at a smaller scale [20]. Two main downscaling approaches are differentiated: dynamical and statistical. Dynamical downscaling refers to the use of regional climate models (RCMs) or limited area models (LAMs) forced laterally or internally by analyses, projections, and simulations of coarser resolution [21]. On the other hand, statistical downscaling relies on methods to transfer large-scale atmospheric variables to smaller-scale variables based on observed local or regional climate data. The large- and small-scale variables are commonly referred to as predictors and predictands, respectively [22]. Once the variables are downscaled, regular and extreme weather events on a smaller spatial and/or temporal scale can be analyzed [23].

Precipitation and temperature are fundamental for addressing climate change and its impacts. One of the reasons is that most historical in-situ observations contain at least these variables. Thus, other meteorological variables are usually derived from precipitation and temperature. In addition, hydrological models and other impact models require a minimum input of precipitation and temperature. Therefore, there are numerous applications for downscaling these variables [24–26]. Nonetheless, limited literature reviews have been performed and mostly compared the performance of different methods in a particular region [27]. Years ago, Maraun [22] compiled techniques to downscale precipitation for climate change studies. Another example is the literature review of downscaling methods for climate change projections where case studies around the world are described [28]. Finally, an assessment of existing approaches and practical considerations to select the best approach for watershed modelling are summarised in a comprehensive review [14].

However, to date and to the authors' knowledge, a systematic review of the downscaling applications comparing their performance in the Andean mountains does not exist. In Europe, a comprehensive framework was established to compare and validate several downscaling methods: VALUE initiative [29], with a regional scope since that is the scale at which climate change impacts are often evaluated. In addition, global climate models are usually downscaled into regional climate models (RCMs) with regional domains.

We propose to study the Andean region due to the challenges in the performance of climate models and downscaling methods in the region. Complex orography causes higher spatial variability of precipitation and temperature. This is true for all mountain regions, but the Andes are particular since they extend from the equatorial tropics to the southern mid-latitudes in a north–south orientation that blocks the main humidity inputs. This creates areas with extremely different climate zones [30].

Another complication in the Andes is the lack of ground stations with continuous records, limiting the outcomes of any downscaling procedure [18]. Therefore, analyzing this region is urgent, since mountains are expected to be more vulnerable to climate change. The Andes only host important ecosystems that provide services such as water supply, water regulation, biodiversity and recreation to 85 million people [31].

Hence, we propose a systematic review in this complex mountainous region to examine the purpose and applications of downscaling methods for precipitation and temperature. In addition, a meta-analysis is included to quantitatively evaluate the performance of these methods. This work is an initial step for further analysis of the climate models used over the region to study climate change projections and their past and future impacts properly and efficiently. The identification of the methods commonly applied for specific applications and regions, as well as those with a better performance in the region will be useful for researchers and decision-makers who can consider the present review as a guiding point to select the proper method or approach for their applications on implementing new models or finding climate projections. Moreover, the review will be highly relevant for the scientific community as it identifies knowledge gaps and untested methods in the countries of the region.

## Objective of the review

This review will summarize the applications and performance for downscaling precipitation and temperature outputs from climate models and reanalysis in the Andean region.

Specifically, we will (i) identify the methods used in each application, (ii) map the exact study area and location of the downscaling application, (iii) describe the purpose and context of each approach, (iv) specify the spatial and temporal resolution of the applications, (v) compile the performance metrics of the application according to the phenomena of interest (when available) (vi) perform the meta-analysis and compare the performance of each method and the factors influencing it, and finally (vii) highlight the most promising methods in terms of fitness for use and identify the untested methods or knowledge gaps in the region.

The systematic review and meta-analysis will describe the performance of the existing downscaling methodologies applied in the Andes and provide information to stakeholders from the region to select the most appropriate downscaling approach for their purpose and study area.

### Primary question

The PICO (Population-Intervention-Comparator-Outcome) framework helps to define the primary review question:

What is the performance of the methods applied in the Andes region for the downscaling of precipitation and temperature outputs from climate models and reanalysis?

To facilitate the review, the question is split into two secondary questions:

1. Which methods are applied in the Andes region for downscaling precipitation and temperature?
2. How do these methods perform in their corresponding study areas?

The components of the primary question are:

- **Population** Climate models and reanalysis datasets in the Andean region containing precipitation and/or temperature outputs.
- **Intervention:** The methods and techniques for downscaling.
- **Comparator:** Observations used as a reference, either gauge-based, satellite-based and or reanalysis.
- **Outcomes:** Performance metrics according to the user problem and phenomena of interest. The metrics or indices used in the studies will depend on the intention or objective of a study. As suggested by the VALUE framework [29], the indices are different for the analysis of extremes, time series, or multivariate aspects. Thus, we have adapted the metrics based on this framework.

For the first secondary question, we mainly require qualitative data as the aim is to identify the methods and purpose of the downscaling procedure. However, the outcomes and performance metrics are very important to answer the second question. There, we will quantify the performance of each approach based on a quantitative synthesis and meta-analysis for those studies reporting the performance metrics mentioned as outcomes.

The 11 performance metrics considered in this review for precipitation and temperature, are described in Table 1.

**Table 1:** Description of the performance metrics considered in this systematic review

#	Performance metric	Abbreviation	Definition	Units
*Marginal aspects (point estimates) of precipitation and temperature*				
1	Relative error or bias of the mean or a particular percentile (5th, 10th, 90th, 95th, other)	RME or AME	Relative or absolute difference between the observed mean or percentile determined from the observation source and the values obtained with the downscaled model (s)	Relative (−), bias in (mm) or (°C)
2	Relative error of the variance of precipitation and/or temperature	RVE	Relative difference between the observed variance and variance obtained with the downscaled model (s)	(-)
3	Relative error/ bias of a specific return level or period (i.e. 20-year, 30-year, 10-season)	RTE or ATE	Relative or absolute difference between the observed value for a specific return period and the value obtained for that same return period with the downscaled model (s)	Relative (−), bias in (mm) or (°C)
4	Bias in the number of threshold exceedances	AEE	Absolute difference between the number of times a threshold is exceeded in the observations and the value obtained with the downscaled model (s)	Threshold in (mm) or (°C) # of exceedances (−)
*Temporal aspects or time series analysis*				
5	Mean squared error	MSE	The average squared difference between the observed values and the downscaled values	(mm^2^) or (°C^2^)
6	Coefficient of determination	R^2^	A number between − 1 and + 1 that measures the strength of the linear relationship between the observed and the downscaled time series	(−)
7	Percentage bias	PBIAS	The average amount that the downscaled numbers is greater or lower than the observed values, expressed as a percentage of the observed value	(%)
8	Bias in the mean (or any percentile) of spell length distribution	ME-SL	The duration of wet/dry spell lengths is analyzed with their distribution. Thus, the index quantifies the absolute difference between the observed mean (or any percentile) and the value obtained with the downscaled model (s)	(# of days)
9	Relative error/bias error of the minimum/maximum of the annual cycle	REMM or AEMM	Relative or absolute difference between the observed maximum/minimum value in the year and the correspondent value obtained with the downscaled model (s)	Relative (−), bias in (mm) or (°C)
*Multivariable aspects (relation between precipitation/temperature with other variables)*				
10	Bias in the Pearson/rank correlation coefficient	ME-PRC	Absolute difference between the Pearson/Rank correlation coefficient in the observations and the downscaled model (s)	(−)
11	Bias in the probability of joint exceedance	ME-PJE	Absolute difference between the observed probability of joint exceedance and the probability from the downscaled model (s)	(−)

## Methods

### Study area

**Fig. 1:**
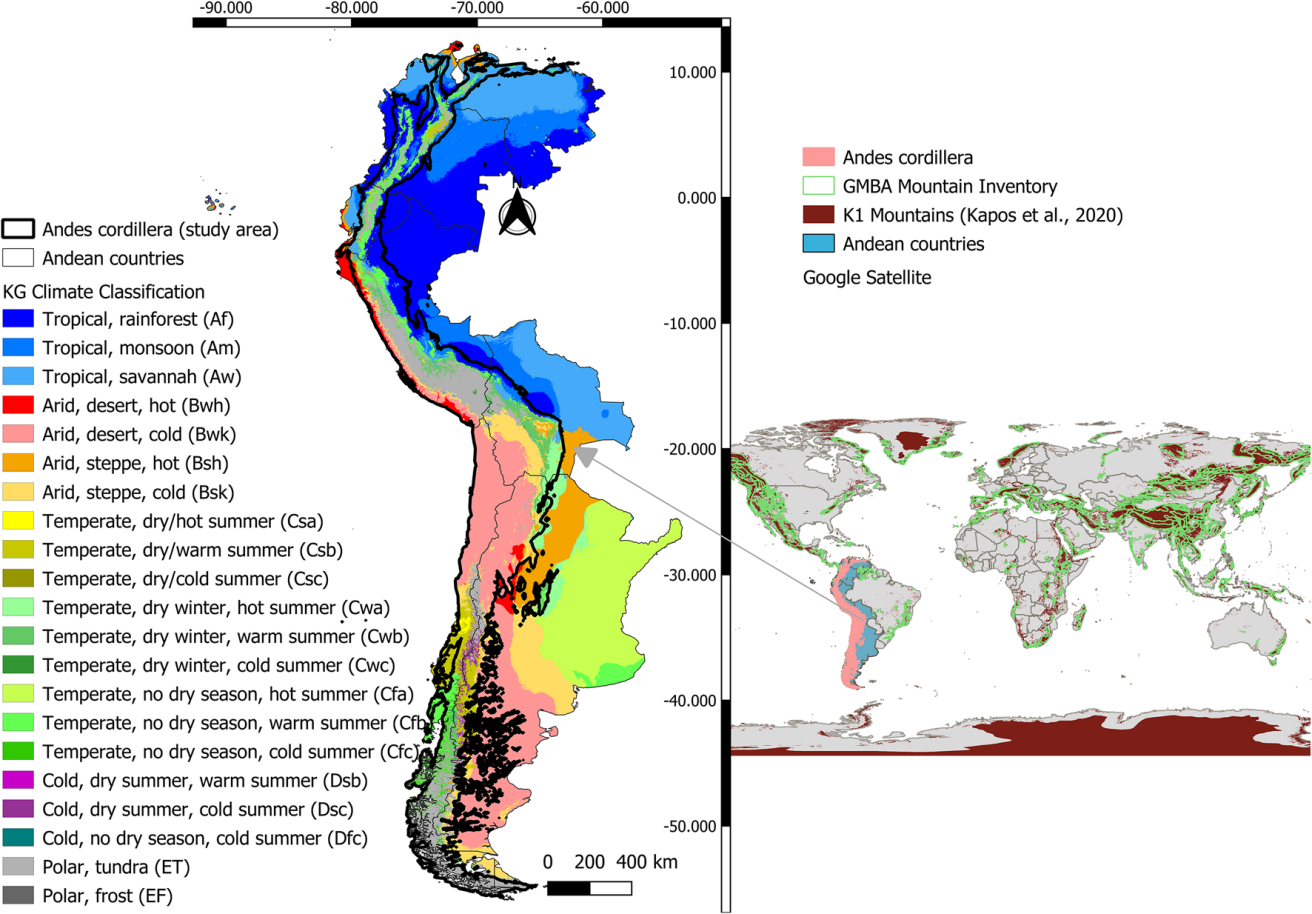
Andean countries polygon with Köppen-Geiger classification [34] (left). Global standards for mountain delimitation (right)

The definition of the Andean region combines two globally accepted standards. First, the South American mountains with the characterization given by [32]. This standard is used to define mountains by the IPCC. Second, the polygon of the Andes Mountain range is obtained from the Global Mountain Biodiversity Assessment (GMBA) according to the definition and criteria proposed by [33]. We remove the mountains from the first criteria located in countries or areas that belong to South America but not to the Andean region. Then, the two layers are merged to include areas in the Andes identified as mountains by the IPCC but excluded from the GMBA polygon or vice versa. Figure 1 shows the polygon of the study area.

#### Systematic review and meta-analysis

In the following sections the term “application” is used to synthesize the procedure of applying a certain downscaling method to a particular climate model or reanalysis to obtain a downscaled time series or downscaled values of particular aspects (point estimates such as the mean, variance or extreme values) of temperature and precipitation. The ROSES form (Additional file 1) is added as supplementary information to demonstrate the reporting of all the methodological details of a systematic review.

#### Identification and engagement of stakeholders

In addition to the multidisciplinary team consisting of experts on water resources, civil engineers, environmental engineers, climatologists, geographers, and data science specialists, conducting the systematic review, relevant stakeholders to this review were identified and engaged. First, the review question and main objectives and limitations of the review have already been discussed and consulted with a group of eleven researchers from different countries in the region with experience in systematic reviews, downscaling and/or climate change projections to ensure the usefulness of this review (see Additional file 2).

### Searching for articles

#### Search languages

Searches will be run using English-language search and include all results in Spanish that appear in the results. In Scopus and WOS databases, at least the abstract of the document is in English and therefore it will be captured. Exceptions are the thesis repository and the grey literature. There we will search in Spanish. As resources are not available and considering we do not expect many articles in other languages, we will limit to English and Spanish.

#### Literature databases and search engines

The review will use the following databases with institutional access of Universidad del Azuay:

1. Scopus.
2. Web of Science (WOS) Core Collection.
3. Scielo.

Preliminary searches returned few documents entirely in Spanish. Therefore, we include an internet search with the Google Scholar engine with a string in Spanish. Due to the affiliation with KU Leuven, the Limo catalog is also proposed. It provides access to the university collection, scientific publications, and scientific articles from leading publishers. However, to avoid bias towards unpublished literature from one university, only published articles are searched.

#### Grey literature

To avoid bias towards published literature, grey literature will be collected from technical reports in Spanish and English of Ministries from each Andean country, National Communications, policies, and plans from supranational organizations such as IPCC and UN related to climate projections. When scientific articles are cited in these reports, the specific articles will be searched and included.

Another source of grey literature are unpublished thesis manuscripts. Due to the enormous amount of university repositories in the Andean countries, a search in each one of these would be impossible. Therefore, we will consider the Latin-American Repository (Universidad de Chile, available at https://repositorioslatinoamericanos.uchile.cl/). It gathers hundreds of repositories from the continent. We will review the thesis of Bachelor, Master and Doctorate degrees to include unpublished but high-quality studies. Once the protocol is published, we will also launch an open invitation through scientific networks (Latin America Early Career Earth System Scientist, Global Young Academy, Science Academies from Andean countries, Ecuadorian Women in Science Network) to compile grey literature or ongoing projects (call for evidence).

Because the grey literature documents may not have been reviewed by international peers, these documents will be classified separately to evaluate the influence of the peer-reviewing process.

#### Other data sources

Additional studies or publications recommended directly by the experience of stakeholders and the review team may also be included. This compilation and the grey literature search will be properly documented and available in the final review.

#### Search terms

The review team conducted a scoping test with several search strings. There will be no time restriction for the publication date. The most recent search was performed in September 2023.

The main search terms are identified:

*Subject:* Ande*, South America, Venezuela*, Colombia*, Ecuador*, Per*, Bolivia*, Chile*, Argentina*

*Intervention:* downscal*, “scale reduction”, wrf*, RegCM*, ARPS*, RCA*, PRECIS, OPM, REM*, ETA*, LAM, “limited area model”, dynamic*, statistical*, regression*, bias*, “delta change”, “quantile mapping”, “machine learning”, “weather generators”, projection*, GCM*, RCM*, “climate model”, “reanalysis”.

*Target:* precipitation*, temperature*, rain*

The asterisk is a wildcard representing any group of characters, including no character. The terms within each category are combined with the Boolean operator “OR”. The categories are combined with the Boolean operator “AND”.

The search strings are detailed in the Additional file 3.

A test list with 73 articles (See Additional file 3) from a previous identification of relevant studies by some members of the review team was used as a benchmark list to estimate the comprehensiveness of the search. With these search terms, 72 out of the 73 studies (98.6%) were retrieved, indicating an optimal search strategy. Moreover, one article was excluded because it focused on glacier mass balance rather than on temperature or precipitation.

### Article screening and study eligibility criteria

#### Screening process

First, duplicated documents will be removed. Then, all collected literature will be filtered according to their title and abstract with the inclusion criteria detailed in the next section. To enhance consistency and avoid mistakes, some articles (at least twenty studies, randomly selected) will be evaluated by all the reviewers to eliminate bias in the inclusion/exclusion criteria. To complement this, a subset of 10% of the articles (around 80) will be screened by four members. In addition, the principal reviewer and another member of the team (at least 2 reviewers) will independently screen all the articles by title, abstract and full-text. The studies rejected based on the title, abstract or full-text assessment will be included in an appendix with the arguments for exclusion. In the same way, the articles whose full text cannot be retrieved will be detailed in the appendix. The entire team will discuss and solve any disagreements between the two reviewers. Review team members will not screen, code or assess study validity of their own papers.

A following screening and classification will be executed to find the literature with enough information to analyze the performance of those methods and extract the metadata and quantitative information about the performance metrics described in Table 1 to pursue the second review question. Also, information on the type of meteorological variable and scale difference considered in the downscaling process will be extracted.

#### Eligibility criteria

The inclusion criteria based on the PICO approach are:

- (Population) The study downscales precipitation and/or temperature outputs from climate models and/or reanalysis in at least one of the 7 Andean countries (Venezuela, Colombia, Ecuador, Perú, Bolivia, Argentina, and Chile) and inside the study area polygon.
- (Intervention) At least one method for spatial downscaling (dynamical and/or statistical) is applied to precipitation and/or temperature outputs from climate models or reanalysis datasets.
- (Comparator) The downscaled series is evaluated against observations (either ground-based, satellite, or reanalysis). If not, then it is only included in the review but not in the meta-analysis.
- (Outcome) When evaluation is carried out, suitable performance metrics for the phenomena of interest are presented.

If there is uncertainty in this stage, the leading reviewer will tend towards inclusion. Then, a full-text screening will consider the following excluding criteria:

- Conference proceedings will be excluded because they normally do not contain enough information about the performance or a complete methodological description.
- Books and book chapters will be excluded because they are usually not related to a specific application or case study that is the scope of this review. However, if a book chapter is based on a published article on a downscaling application in the Andean region, then the article will be included.

#### Data extraction and coding

An Excel spreadsheet is considered the main database. Due to the number of expected studies, we foresee the use of the open-access software CADIMA (Julius Kühn-Institute). This tool allows for more efficient cooperation among the review team.

##### a. Metadata

Once the full-text articles are retrieved, the review team will extract the data. First, the descriptive information and the metadata from each article. Among these data are:

- a.1 Author.
- a.2 Type of literature: published article, review, grey literature-thesis, grey literature-official or institutional document.
- a.3 Journal or Institution.
- a.4 Reference.
- a.5 Publication year.
- a.6 Abstract: paste the entire abstract.
- a.7 Study area, country (ies) and city (ies).
- a.8 Climatic regions, eco-region or ecosystem: e.g.: choco, pampa, altiplano. The GMBA inventory definition is used as a reference [33].
- a.9 Geographical coordinates: bounding box (upper left corner; down right corner) or if it is a point application, then just the coordinates.
- a.10 Elevation range of the study area: maximum and minimum elevation.
- a.11 Climate features: rainfall regime (Unimodal/bimodal/trimodal, yearly rainfall, month with the highest and lowest monthly average).
- a.12 Climate features: temperature patterns (Range of variation during the year, warmest and coldest months).
- a.13 Climate region according to the Köppen-Geiger classification [34].
- a.14 Dominant large-scale or local process influencing the climate in the region.

##### b. Coding

This review intends to map the exact study area and location of the downscaling application. Coding facilitates this process and the identification. Here, the code will be assigned to each application in the study based on four categories: country, downscaling method, purpose and variable of interest. Note that the code is assigned to each application and not per study. The codes are:

i. Country: Venezuela (VE), Colombia (CO), Ecuador (EC), Peru (PE), Bolivia (BO), Chile (CH), Argentina (AR).
ii. Downscaling method: statistical downscaling (SD), dynamical downscaling (DD).
iii. Purpose of the application: Climate change (CC), evaluation (EV), process understanding (PU), others (O).
iv. Variable of interest/predictand: precipitation (P), mean temperature (T), maximum temperature (TX), minimum temperature (TM).In the end, a number accounts for similar applications to avoid duplication (where the four items coincide). This number uses the date of the conclusion of the study. A study splits into various applications when it evaluates more than one variable or in different locations.

The use of codes allows for easy filtering and grouping. For instance, if an application corresponds to an application using a statistical downscaling method to downscale the precipitation from a climate model to evaluate the impacts of climate change in Ecuador, then the code would be:

EC-SD-CC-P-001

Which would stand for Ecuador – Statistical Downscaling – Climate Change – Precipitation – Study number 1.

##### c. Full data extraction

In addition to the meta-data, a full data extraction from the materials, results, and conclusions will be carried out. We will compile both quantitative and qualitative information. It is important to mention that the critical appraisal (next section) is executed while performing this task.

##### d. Qualitative data

The study splits into several rows if it evaluates more than one model or method. The data is extracted for the intervention, comparator and outcome as follows.

Intervention:

- d.1 Variable of interest: precipitation and/or minimum, maximum, mean temperature.
- d.2 Dynamical model.
- d.3 Statistical method.
- d.4 External forcing: GCM, RCM, Reanalysis.
- d.5 Generation of the climate model (CMIP5, CMIP6, etc)
- d.6 Parametrizations (dynamical downscaling).
- d.7 Predictors (statistical downscaling).
- d.8 Use or purpose of the downscaling method (calibration, evaluation, forecasting, climate change projections, process understanding).

Comparator:

- d.9 Observation datasets or products used as a reference for calibration/validation of downscaling methods.
- d.10 Historical or control period.
- d.11 Climate change scenarios (number and which).
- d.12 Methodology used for calibration.
- d.13 Methodology used for validation or evaluation.

Outcomes:

- d.14 Qualitative evaluation of the results (as stated in the paper).
- d.15 Work needed further (as stated in the paper).
- d.16 Boolean: Is the study comparing the downscaling application (treatment) versus the original reanalysis/model/data prior to downscaling (control-without intervention)? (Yes/No).

##### e. Quantitative data

Intervention:

- e.1 Spatial resolution (km), before and after downscaling.
- e.2 Temporal resolution (daily, monthly, etc.), before and after downscaling.
- e.3 Number of GCMs or reanalysis sets (detail them).

Comparator:

- e.4 Years used as historical/base/control period with the observations (base duration).
- e.5 Years used as validation or evaluation period.
- e.6 Years covered by the projections (future duration).

Outcomes.

- e.7 Performance metrics of the control group in the evaluation/validation period (include units). One row for each performance metric used in the study and listed in Table 1.
- e.8 Performance metrics of the treatment group in the evaluation/validation period.

An example data extraction spreadsheet is available as Additional file 4 for clarity and transparency.

If a study does not provide or present the information, then “Not Stated” will be used. Whereas if the information required does not apply to that particular study, then the table is filled with “Not Applicable or N/A”. The articles will be divided into even groups to allow cooperation among authors in the data extraction. Twenty of the articles will be common and will be analyzed by all the team to assess replicability in the data extraction. In addition, the lead reviewer will extract data from all the articles to verify and ensure consistency. The entire team will discuss and solve any disagreements between the reviewers.

All the extracted data records and databases will be made available as additional files.

## Study validity assessment

As recommended by CEE guidelines, a critical appraisal step is needed to reduce the influence of the potential risk of bias in each study. Here, we will test two validity types: internal validity and external validity. The former is related to the methodological design of the research and the potential risk of bias. The latter provides an idea of how applicable and generalizable is for the review question.

To the author´s knowledge, there is no available tool to perform a critical appraisal for downscaling studies. Thus, the review team has developed a new criterion based on preliminary meetings with stakeholders, reviewers’ suggestions and literature [38]. Guiding questions aim to evaluate the risk of bias of each study, with seven questions focused on internal validity and systematic bias due to confounding, selection, performance risk, missing data, reporting bias or statistical errors. The last three questions are oriented to identify studies with low external validity. A checklist is proposed to register the answer of each study (see Additional file 5). If an answer to any question is NO, the reviewer must explain the rationale behind this risk of bias. The questions are:

- Is the comparator a suitable reference observation (ground-based observations, reanalysis, validated satellite product)?
- Have any sensitivity analyses been conducted to explore uncertainty around the model outputs (e.g. varying input data, parameterizations, or testing the main assumptions)?
- Was the validation period selected in an adequate and non-arbitrary way (i.e. random sample of years, time split validation, cross-validation for machine-learning methods)?
- Have the authors avoided arbitrary procedures or corrections to artificially improve the performance of the downscaling method?
- Have the authors considered a quality check of the climate data (i.e. missing data, homogeneity)?
- Are the performance metrics reported for all the evaluated points and not only an arbitrary portion of them?
- Are the statistical calculations correct and without obvious limitations or errors?
- Was the downscaling method evaluated against two or more observation points, to account for spatial replicability?
- Are the assumptions of the method valid and appropriate in other regions (can it be generalised)?
- Is the downscaling method and the statistical analysis coded in an open-source tool or a well-documented software that can be used to reproduce and verify the analysis?

When the answer to all these questions is YES, then the study is judged to have LOW risk of bias. In contrast, when the answer to at least one question is NO, then the study will be judged to have a HIGH risk of bias. When there is no information to answer one of these questions but none of them have been answered with NO, then the study will be judged to have an UNCLEAR risk of bias.

The appraisal of each study will be performed by two reviewers. In addition, at least ten percent of the articles will be appraised and graded by all the members of the reviewing team to check the consistency of this step. A member of the reviewing team will not critically appraise a document in which he or she has participated.

### Potential reasons for heterogeneity or effect modifiers

To understand the differences in the performance of the downscaling methods, a short list of factors causing heterogeneity and affecting the performance was compiled based on the expertise of the review team and consultation with the stakeholders. Some of these variables will be used as subgroups and the last five will be incorporated as explanatory variables in meta-regression.

- Generation of the GCM or RCM (e.g. CMIP4, CMIP5, CMIP6).
- Dominant large-scale processes influencing the climate (e.g. ENSO, PDO, orographic forcing).
- Climate zone or ecoregion.
- Elevation range (maximum and minimum elevation)
- Predictors used (only in statistical downscaling).
- Parametrizations used (only in dynamical downscaling).
- Temporal resolution before and after downscaling.
- Validation method (i.e. alternate years, control and base model, future “control”, pseudo-reality, cross-validation).
- Observation dataset type (gauge-based, reanalysis, satellite-based).
- Climate conditions of the region (e.g. mean and variance of the precipitation and temperature).
- Spatial resolution of the driving climate model or reanalysis.
- Latitude.
- Longitude.
- Duration of the calibration period.
- Duration of the evaluation/validation period.

### Meta-analysis

The performance of the downscaling methods is summarized with the performance metrics described in Sect. 4. However, due to resource limitations, only the most recurrent metrics (the top three most reported by studies) will be considered in the meta-analysis. The logarithm of response ratio (lnRR) will be calculated for each performance metric as effect size in the meta-analysis. The measure is chosen as it is unitless and allows for comparison between groups. This ratio between means is calculated with Eq. 1.

1 $$lnRR={\text{ln}}\left(\frac{{X}_{t}}{{X}_{c}}\right)$$

Here, X represents the mean of the performance metric, and the suffixes c and t correspond to the control and treatment respectively. The studies that will be included in this section of the review, require a paired comparison between the performance of the original dataset (reanalysis, climate model, satellite data) prior to downscaling (control) and the performance of the downscaled model (treatment/after intervention). When studies do not report the performance metrics of the original dataset, reviewers will contact the authors to complete the data.

The three effect sizes (log-response ratios of the three most recurrent performance metrics) will be included separately in three different meta-analytic models. Multilevel meta-analytic models will be fitted to estimate the overall effect size of each downscaling method to avoid non-independence issues [35].

The implementation is foreseen with the packages *metafor* [36] and *metagear* [40] which have functions for conducting meta-analyses in *R* [39]. Then, relative heterogeneity between studies will be estimated using the I-square statistic test. The magnitude of I^2^ may also inform which predictor variable is likely to explain the heterogeneity and used in the meta-regression. In addition, prediction intervals (95%) will be added as a complementary method to show the predicted range of values and visualize the heterogeneity in orchard plots [35].

To explain part of the between-study heterogeneity, the three effect sizes will be plotted against categorical moderators (effect modifiers). The classification of the results by the downscaling method aims to identify patterns or clusters due to the influence in the performance of modifiers in each method.

Finally, meta-regressions are proposed to explain the heterogeneity and to identify possible relations between the three chosen performance metrics (i.e. effect sizes once they are converted to lnRR) and quantitative variables identified as possible sources of heterogeneity, such as: spatial resolution of the climate model or reanalysis before the downscaling, duration of the calibration, duration of the validation, latitude, longitude and elevation range.

Additionally, where some metrics are mentioned in the evaluation but not reported, the review team will try to contact the authors or perform basic calculations to complete the results. A more detailed explanation of the proposed meta-analysis is detailed in Additional file 6.

### Influence of the validity of the studies and publication bias

A sensitivity analysis will be conducted in the meta-analysis by including and excluding the applications identified as low-validity (High risk of bias) during the critical appraisal. A sensitivity analysis will be used as well by presenting results including and excluding grey literature.

Finally, graphical tests using funnel plots (effect size against standard error or sample size) are proposed to explore the small study effect. Moreover, multilevel meta-regressions will test for the small study effect and the decline (time-lag) effect.

### Data synthesis and presentation

All the steps of this systematic review will be synthesized in a flow diagram indicating the number of studies included in each stage, following the Roses template [37]. Then, three syntheses are proposed in the review: narrative, quantitative and meta-analysis.

The narrative synthesis of data and results from all the studies in this review will describe the quality and quantity of the available evidence, as well as the performance of the downscaling methods in the Andean region. The applications will be summarized in tables describing their methods and purposes. Then, the location of the different studies will be presented on a map. This enhances the identification of knowledge clusters and gaps.

The quantitative synthesis will aggregate the applications by variable, country, method, resolution and purpose. Therefore, we suggest heatmaps to graphically display the number of applications within a certain group. Finally, the meta-analysis will be presented with tables, forest plots for the three most reported performance metrics converted to log-response ratios and meta-regressive plots. It is proposed to present results using orchard plots as recommended by (57) because they can present results across different groups and categories. The systematic review will end with a discussion highlighting the most promising methods for downscaling temperature and precipitation for the region. Also, the strengths and weaknesses of existing studies, the main factors influencing the performance, and knowledge gaps or untested methods in the region.

## Supplementary Information

**Additional file 1.** Roses form.

**Additional file 2.** Affiliations and field of expertise of the involved stakeholders.

**Additional file 3.** Search strings and benchmark list.

**Additional file 4.** Data coding and extraction template.

**Additional file 5.** Critical appraisal checklist.

**Additional file 6.** Meta-analysis strategy.

## Data Availability

All material will be provided upon request.
