# Supplementary material for: Downscaling precipitation and temperature in the Andes: applied methods and performance—a systematic review protocol
Source: Environ Evid. 2023 Dec 12;12:29. doi: 10.1186/s13750-023-00323-0 (PMC11378818; doi:10.1186/s13750-023-00323-0)
Supplement: Supplementary file 1 — Additional file 1. Roses form. [file 13750_2023_323_MOESM1_ESM.pdf]

Item number	Section / sub-section	Topic	Description	Further explanation	Checklist/Meta-data	Author response	Comments
1	Title	Title	The title must indicate that it is a systematic review protocol, and must indicate if it is an update/amendment: e.g. "A systematic review update protocol...".	The title should normally be the same or very similar to the review question.	Meta-data	Downscaling precipitation and temperature in the Andes. Applied methods and performance. A systematic review protocol.	
2	Type of review	Type of review	Select one of the following types of review: systematic review, systematic	See CEE Guidance on amendments and updates [1]	Meta-data	systematic review	
3	Authors contacts	Authors contacts	The full names, institutional addresses, and email addresses for all authors		Checklist	Yes	
4	Abstract	Structured summary	Abstract must not exceed 350 words and must include two sections 1)		Checklist	Yes	
5	Background	Background	Describe the rationale for the review in the context of what is already	A theory of change and/or conceptual model can be presented that links	Checklist	Yes	
6	Stakeholder engagement	Stakeholder engagement	The planned/actual role of stakeholders throughout the review process (e.g.		Checklist	Yes	
7	Objective of the review	Objective	Describe the primary question and secondary questions (when applicable).	The primary question is the main question of the review. Secondary	Checklist	Yes	
8		Definitions of the question components	Break down and summarise question key elements e.g. population, intervention(s)/exposure(s), comparator(s), and outcome(s).	For other question types see [3,4]	Meta-data	**Population:** Climate models and reanalysis datasets in the Andean region containing precipitation and/or temperature outputs. **Intervention:** The methods and techniques for downscaling. **Comparator:** Observations used as reference, either gauge-based, satellite-based and or reanalysis. **Outcomes:** Performance metrics depending on the user problem and the phenomena of interest.	
	Methods						
9	Searches	Search strategy	Detail the planned search strategy to be used, including: database names	Details regarding search strategy testing should be provided.	Checklist	Yes	
10		Search string	Provide Boolean-style full search string and state the platform for which the string is formatted (e.g. Web of Science format)		Meta-data	TITLE-ABS-KEY ((Ande* OR ((mountain* or highland*) and (“South America” OR Venezuela* OR Colombia* OR Ecuador* OR Peru* OR Bolivia* OR Chile* OR Argenti*))) AND (rain* OR precipitation OR temperature) AND (RCM* OR “regional climate model” OR downscal* OR “scale reduction” OR wrf* OR RegCM* OR ARPS OR RCA OR PRECIS OR OPM OR REMO OR ETA OR LAM OR “limited area model” OR (downscal* and (“bias correction” OR “delta change” OR “quantile mapping” OR “weather generators” OR “weather typing” OR “quantile perturbation” OR dynamic* OR statistical* OR regression* OR “machine learning”)) OR (downscal* and (GCM* OR “circulation model” OR “global climate model” OR “reanalysis” OR projection*))))	Only one search string. The others are in the manuscript
11		Languages – bibliographic databases	List languages to be used in bibliographic database searches.		Meta-data	Spanish, English	
12		Languages – grey literature	List languages to be used in organizational websites searches and web-		Meta-data	Spanish, English	
13		Bibliographic databases	Provide the number of bibliographic databases to be searched.		Meta-data	4	Scielo, WOS Core Collection, Scopus, KU Leuven LIMO
14		Web – based search engines	Provide the number of web – based search engines to be searched.		Meta-data	1	Google scholar
15		Organisational websites	Provide the number of organisational websites to be searched.		Meta-data	8	Latin american thesis repository and other websites from ministries, NGOs and others (at least 1 per country)
16		Estimating the comprehensiveness	Describe the process by which the comprehensiveness of the search		Checklist	Yes	
17		Search update	Describe any plans to update the searches during the conduct of the review.	Optional. A search update is good practice if original searches were	Checklist	n/a	Last update in September 2023
18	Article screening and study inclusion	Screening strategy	Describe the methodology for screening articles/studies for		Checklist	Yes	
19		Consistency checking	Describe clearly the process for checking consistency of decisions including		Checklist	Yes	
20		Inclusion criteria	Describe the inclusion criteria used to assess relevance of identified		Checklist	Yes	
21		Reasons for exclusion	State that you will provide a list of articles excluded at full text with reasons		Checklist	Yes	
22	Critical appraisal	Critical appraisal	Describe here the method you propose for critical appraisal of study validity		Checklist	Yes	
23		Critical appraisal strategy	Describe how the information from critical appraisal will be used in		Checklist	Yes	
24		Consistency checking	Describe how repeatability of critical appraisal of study validity will be		Checklist	Yes	
25	Data extraction	Meta-data extraction and coding	Describe the method for meta-data extraction and coding for studies		Checklist	Yes	
26		Data extraction strategy	Describe the method for extraction of qualitative and/or quantitative study		Checklist	Yes	
27		Approaches to missing data	Describe any processes for obtaining and confirming missing or unclear		Checklist	Yes	
28		Consistency checking	Describe how repeatability of the meta-data/data extraction process will be		Checklist	Yes	
29	Potential effect modifiers/reasons	Potential effect modifiers/reasons	Provide a list of and justification for the effect modifiers /reasons for	The list should not be exhaustive but a short list of those variables thought	Checklist	Yes	
30	Data synthesis and presentation	Data synthesis and presentation	State the type of synthesis conducted as part of the systematic review		Meta-data	Narrative and quantitative. With meta-analysis	
31		Narrative synthesis strategy	Describe methods to be used for narratively synthesising the evidence base	Vote-counting (tallying of studies based on the direction or significance of	Checklist	Yes	
32		Quantitative synthesis strategy	If data are appropriate for quantitative synthesis, describe planned	Compulsory if appropriate for data	Checklist	Yes	
33		Qualitative synthesis strategy	Describe methods to be used for synthesising qualitative data and justify	Compulsory if appropriate for data	Checklist	Yes	
34		Other synthesis strategies	Describe any other approaches to be used for synthesising data or	Compulsory if appropriate for data	Checklist	Yes	
35		Assessment of risk of publication	Describe planned methods for examining the possible influence of	For quantitative syntheses this may be done using diagnostic plots or	Checklist	Yes	
36		Knowledge gap identification	Describe the methods to be used to identify and/or prioritise key	Optional	Checklist	Yes	
37		Demonstrating procedural	Describe the role of systematic reviewers (who have also authored articles	Reviewers who have authored articles to be considered within the review	Checklist	Yes	
38	Declarations	Competing interests	Describe of any financial or non-financial competing interests that the		Checklist	Yes	

References

[1] Bayliss, H.R., Haddaway, N.R., Eales, J., Frampton, G.K. and James, K.L., 2016. Updating and amending systematic reviews and systematic maps in environmental management. Environmental Evidence, 5(1), p.20.

[2] Haddaway, N.R., Kohl, C., da Silva, N.R., Schiemann, J., Spök, A., Stewart, R., Sweet, J.B. and Wilhelm, R., 2017. A framework for stakeholder engagement during systematic reviews and maps in environmental management. *Environmental Evidence* , *6* (1), p.11.

[3] Collaboration for Environmental Evidence. 2018. Guidelines and Standards for Evidence synthesis in Environmental Management. Version 5.0. www.environmentalevidence.org/information-for-authors.

[4] Leeds Institute of Health Sciences. https://medhealth.leeds.ac.uk/info/639/information_specialists/1500/search_concept_tools. Accessed 12/11/2017.
