# Supplementary material for: Downscaling precipitation and temperature in the Andes: applied methods and performance—a systematic review protocol
Source: Environ Evid. 2023 Dec 12;12:29. doi: 10.1186/s13750-023-00323-0 (PMC11378818; doi:10.1186/s13750-023-00323-0)
Supplement: Supplementary file 2 — Additional file 2. Affiliations and field of expertise of the involved stakeholders. [file 13750_2023_323_MOESM2_ESM.docx]

**README**

This file presents the affiliations and field of expertise of the stakeholders involved informally during the preliminary stage of the review to evaluate the main objective, applicability, and limitations of this review.

Additional File 2: Affiliations of involved stakeholders during the preliminary stage (main objective and limitations of the review) and field of expertise.

1. Universidad Nacional de Colombia. Country: Colombia. Field of expertise: Generation of climate change projections under climate change scenarios. Numerical modelling of weather and climate.
2. Universidad de Cuenca. Country: Ecuador. Field of expertise: Hydrological models, environmental applications, and use of downscaling for climate change impact studies.
3. Universidad de Cuenca. Country: Ecuador. Field of expertise: Mathematical modelling of climate and hydrological processes. Data-driven techniques.
4. UNDP. United Nations Development Programme. Country: Ecuador. Field of expertise: Adaptation planning based on climate change projections. Project management and consultant.
5. Universidad Nacional San Cristóbal de Huamanga – Universidade de Sao Paulo. Country: Peru. Field of expertise: Climate change and natural hazards. Dynamical and statistical downscaling.
6. Universidad Nacional Autónoma de México. Country: México. Field of expertise: Groundwater modelling. Climate change impacts on aquifers recharge.
7. Ministry of Environment and Ecological Transition. Country: Ecuador. Field of expertise: Climate change adaptation policies and climate risk consultancies.
8. Universidad del Azuay. Country: Ecuador. Field of expertise: Medical science. Valuable experience in systematic reviews and meta-analysis. Methodological aspects of systematic reviews.
9. Stockholm University. Country: Sweden. Field of expertise: Hydrology and freshwater resources. Climate change impacts.
10. Universidad del Rosario. Country: Colombia. Field of expertise: Analysis of climate change’s impacts on the paramo (tropical alpine) vegetation based on climate models.
11. Universidad Mayor de San Simón. Country: Bolivia. Field of expertise: Hydrology and Climate change.
