# Supplementary material for: Downscaling precipitation and temperature in the Andes: applied methods and performance—a systematic review protocol
Source: Environ Evid. 2023 Dec 12;12:29. doi: 10.1186/s13750-023-00323-0 (PMC11378818; doi:10.1186/s13750-023-00323-0)
Supplement: Supplementary file 3 — Additional file 3. Search strings and benchmark list. [file 13750_2023_323_MOESM3_ESM.docx]

**README**

This file presents the search strings used in the review for each of the databases.

In addition, it includes the test list with 73 articles (Benchmark list) that was used to estimate the comprehensiveness of the search. With these search terms, 72 out of the 73 studies (98.6%) should be returned. Only Moller et al., 2010 was not retrieved because it focused on glacier mass balance rather than on precipitation and temperature. Thus, it was excluded.

**Search Strings**

**Google Scholar (sorted by relevance n=100) -To evaluate the chance of missing documents entirely in Spanish.**

*with all of the words: downscaling reducción de escala andes clim*

**Scielo**

English:

(downscal*) AND (clim*) AND (andes OR venezuela OR colombia OR ecuador OR peru OR bolivia OR argentina OR chile)

Spanish:

((reducción de escala) OR (dowscaling)) AND ((andes) OR (venezuela) OR (colombia) OR (ecuador) OR (peru) OR (bolivia) OR (chile) OR (argentina)) AND ((lluvia) OR (temperatura) OR (precip))

**Scopus**

- TITLE-ABS-KEY ((Ande* OR “South America” OR Venezuela* OR Colombia* OR Ecuador* OR Peru* OR Bolivia* OR Chile* OR Argenti*) AND (rain* OR precipitation OR temperature) AND (RCM* OR “regional climate model” OR downscal* OR “scale reduction” OR wrf* OR RegCM* OR ARPS OR RCA OR PRECIS OR OPM OR REMO OR ETA OR LAM OR “limited area model” OR (downscal* and (“bias correction” OR “delta change” OR “quantile mapping” OR “weather generators” OR “weather typing” OR “quantile perturbation” OR dynamic* OR statistical* OR regression* OR “machine learning”)) OR (downscal* and (GCM* OR “circulation model” OR “global climate model” OR “reanalysis” OR projection*))))

**WoS Core Collection**

- TS=((Ande* OR “South America” OR Venezuela* OR Colombia* OR Ecuador* OR Peru* OR Bolivia* OR Chile* OR Argenti*) AND (rain* OR precipitation OR temperature) AND (RCM* OR “regional climate model” OR downscal* OR “scale reduction” OR wrf* OR RegCM* OR ARPS OR RCA OR PRECIS OR OPM OR REMO OR ETA OR LAM OR “limited area model” OR (downscal* and (“bias correction” OR “delta change” OR “quantile mapping” OR “weather generators” OR “weather typing” OR “quantile perturbation” OR dynamic* OR statistical* OR regression* OR “machine learning”)) OR (downscal* and (GCM* OR “circulation model” OR “global climate model” OR “reanalysis” OR projection*))))

**KU Leuven Limo (applying the filter: “only articles” to exclude other resources available in the repository)**

- Title or subject or description containing: ((Ande* OR ((mountain* OR highland*) AND (“South America” OR Venezuela* OR Colombia* OR Ecuador* OR Peru* OR Bolivia* OR Chile OR Argenti*))) AND (rain* OR precipitation OR temperature) AND (RCM* OR “regional climate model” OR downscal* OR “scale reduction” OR wrf* OR RegCM* OR ARPS OR RCA OR PRECIS OR OPM OR REMO OR ETA OR LAM OR “limited area model” OR (downscal* AND (“bias correction” OR “delta change” OR “quantile mapping” OR “weather generators” OR “weather typing” OR “quantile perturbation” OR dynamic* OR statistical* OR regression* OR “machine learning”)) OR (downscal* AND (GCM* OR “circulation model” OR “global climate model” OR “reanalysis” OR projection*))))

**Repositorio Latinoamericano de tesis (search by title and subject), ignore projects and conference proceedings**

The search terms are: (downscal* OR escala) + clim*

**Preliminary results (after scoping) with these search terms:**

- Google Scholar = Only the first 100 results sorted by relevance
- SCOPUS = 704 results
- WOS Core Collection = 831 results
- KU Leuven Limo (articles) = 455 results
- Scielo = 11 results (English) and 10 results (Spanish)

Total = 2111 results (before duplicates removal)

After duplicates removal = 1412

Preliminary grey literature

- Repositorio latinoamericano de tesis = 41 thesis results
- Other sources (governments, other organizations, reports) = 12

**Test list with 73 articles (Benchmark list)**

1.

Alexander P, Ruscica R, Sörensson AA, Menéndez CG. Gravity wave momentum flux generation close to mid-latitude Andes in mesoscale simulations of late 20th and 21st centuries. Advances in Space Research. 2011 Oct;48(8):1359–70.

2.

Alvial Vásquez FJ, Abarca-del-Río R, Ávila AI. High-Resolution Precipitation Gridded Dataset on the South-Central Zone (34° S–41° S) of Chile. Front Earth Sci. 2020 Oct 29;8:519975.

3.

Araya-Osses D, Casanueva A, Román-Figueroa C, Uribe JM, Paneque M. Climate change projections of temperature and precipitation in Chile based on statistical downscaling. Clim Dyn. 2020 May;54(9–10):4309–30.

4.

Avilés A, Palacios K, Pacheco J, Jiménez S, Zhiña D, Delgado O. Sensitivity exploration of water balance in scenarios of future changes: a case study in an Andean regulated river basin. Theor Appl Climatol. 2020 Aug;141(3–4):921–34.

5.

Barrett BS, Garreaud RD, Falvey M. Effect of the Andes Cordillera on precipitation from a midlatitude cold front. Monthly Weather Review. 2009;137(9):3092–109.

6.

Bendix J, Trachte K, Palacios E, Rollenbeck R, Göttlicher D, Nauss T, et al. El Niño meets La Niña-anomalous rainfall patterns in the “traditional” El Niño region of Southern Ecuador. Erdkunde. 2011;65(2):151–67.

7.

Bozkurt D, Rojas M, Boisier JP, Valdivieso J. Projected hydroclimate changes over Andean basins in central Chile from downscaled CMIP5 models under the low and high emission scenarios. Climatic Change. 2018 Oct;150(3–4):131–47.

8.

Bozkurt D, Rojas M, Boisier JP, Rondanelli R, Garreaud R, Gallardo L. Dynamical downscaling over the complex terrain of southwest South America: present climate conditions and added value analysis. Clim Dyn. 2019 Dec;53(11):6745–67.

9.

Buytaert W, Vuille M, Dewulf A, Urrutia R, Karmalkar A, Célleri R. Uncertainties in climate change projections and regional downscaling in the tropical Andes: implications for water resources management. Hydrol Earth Syst Sci. 2010 Jul 15;14(7):1247–58.

10.

Cabré F, Solman S, Núñez M. Climate downscaling over southern South America for present-day climate (1970-1989) using the MM5 model. Mean, interannual variability and internal variability. Atmósfera. 2014 Apr;27(2):117–40.

11.

Campozano L, Tenelanda D, Sanchez E, Samaniego E, Feyen J. Comparison of Statistical Downscaling Methods for Monthly Total Precipitation: Case Study for the Paute River Basin in Southern Ecuador. Advances in Meteorology. 2016;2016:1–13.

12.

Chimborazo O, Vuille M. Present-day climate and projected future temperature and precipitation changes in Ecuador. Theoretical and Applied Climatology. 2021;143(3–4):1581–97.

13.

De La Torre A, Alexander P, Hierro R, Llamedo P, Rolla A, Schmidt T, et al. Large-amplitude gravity waves above the southern Andes, the Drake Passage, and the Antarctic Peninsula. Journal of Geophysical Research Atmospheres. 2012;117(2).

14.

de la Torre A, Hierro R, Llamedo P, Rolla A, Alexander P. Severe hailstorms near Southern Andes in the presence of mountain waves. Atmospheric Research. 2011;101(1–2):112–23.

15.

De Sales F, Xue Y. Investigation of seasonal prediction of the South American regional climate using the nested model system. J Geophys Res. 2006 Oct 28;111(D20):D20107.

16.

Eghdami M, Barros AP. Extreme orographic rainfall in the eastern Andes tied to cold air intrusions. Frontiers in Environmental Science. 2019;7(JUL).

17.

Escanilla-Minchel R, Alcayaga H, Soto-Alvarez M, Kinnard C, Urrutia R. Evaluation of the Impact of Climate Change on Runoff Generation in an Andean Glacier Watershed. Water. 2020 Dec 17;12(12):3547.

18.

Exbrayat JF, Buytaert W, Timbe E, Windhorst D, Breuer L. Addressing sources of uncertainty in runoff projections for a data scarce catchment in the Ecuadorian Andes. Climatic Change. 2014 Jul;125(2):221–35.

19.

Fiebig-Wittmaack M, Astudillo O, Wheaton E, Wittrock V, Perez C, Ibacache A. Climatic trends and impact of climate change on agriculture in an arid Andean valley. Climatic Change. 2012 Apr;111(3–4):819–33.

20.

Flores-Rojas JL, Moya-Alvarez AS, Kumar S, Martinez-Castro D, Villalobos-Puma E, Silva-Vidal Y, et al. Analysis of Possible Triggering Mechanisms of Severe Thunderstorms in the Tropical Central Andes of Peru, Mantaro Valley. ATMOSPHERE. 2019 Jun;10(6).

21.

Flores-Rojas JLL, Moya-Álvarez ASS, Valdivia-Prado JMM, Piñas-Laura M, Kumar S, Karam HAA, et al. On the dynamic mechanisms of intense rainfall events in the central Andes of Peru, Mantaro valley. Atmospheric Research. 2021;248.

22.

Garreaud RD, Fuenzalida HA. The influence of the Andes on cutoff lows: A modeling study. Monthly Weather Review. 2007;135(4):1596–613.

23.

Guevara Luna MA, Casallas A, Belalcázar Cerón LC, Clappier A. Implementation and evaluation of WRF simulation over a city with complex terrain using Alos-Palsar 0.4 s topography. Environmental Science and Pollution Research. 2020;27(30):37818–38.

24.

Hellström RÅ, Fernández A, Mark BG, Michael Covert J, Rapre AC, Gomez RJ. Incorporating Autonomous Sensors and Climate Modeling to Gain Insight into Seasonal Hydrometeorological Processes within a Tropical Glacierized Valley. Annals of the American Association of Geographers. 2017 Mar 4;107(2):260–73.

25.

Heredia MB, Junquas C, Prieur C, Condom T. New Statistical Methods for Precipitation Bias Correction Applied to WRF Model Simulations in the Antisana Region, Ecuador. Journal of Hydrometeorology. 2018 Dec 1;19(12):2021–40.

26.

Hofer M, Marzeion B, Mölg T. A statistical downscaling method for daily air temperature in data-sparse, glaciated mountain environments. Geosci Model Dev. 2015 Mar 12;8(3):579–93.

27.

Hofer M, Nemec J, Cullen NJ, Weber M. Evaluating Predictor Strategies for Regression-Based Downscaling with a Focus on Glacierized Mountain Environments. Journal of Applied Meteorology and Climatology. 2017 Jun;56(6):1707–29.

28.

Hoyos CD, Ceballos LI, Pérez-Carrasquilla JS, Sepulveda J, López-Zapata SM, Zuluaga MD, et al. Meteorological conditions leading to the 2015 Salgar flash flood: Lessons for vulnerable regions in tropical complex terrain. Natural Hazards and Earth System Sciences. 2019;19(11):2635–65.

29.

Jaeschke A, Boehm C, Merklinger FF, Bernasconi SM, Reyers M, Kusch S, et al. Variation in delta N-15 of fog-dependent Tillandsia ecosystems reflect water availability across climate gradients in the hyperarid Atacama Desert. Vol. 183, GLOBAL AND PLANETARY CHANGE. RADARWEG 29, 1043 NX AMSTERDAM, NETHERLANDS: ELSEVIER; 2019.

30.

Jimenez-Sanchez G, Markowski PM, Jewtoukoff V, Young GS, Stensrud DJ. The Orinoco Low-Level Jet: An Investigation of Its Characteristics and Evolution Using the WRF Model. JOURNAL OF GEOPHYSICAL RESEARCH-ATMOSPHERES. 2019 Oct;124(20):10696–711.

31.

Junquas C, Takahashi K, Condom T, Espinoza JC, Chavez S, Sicart JE, et al. Understanding the influence of orography on the precipitation diurnal cycle and the associated atmospheric processes in the central Andes. Climate Dynamics. 2018;50(11–12):3995–4017.

32.

Kiefer J, Karamperidou C. High‐Resolution Modeling of ENSO‐Induced Precipitation in the Tropical Andes: Implications for Proxy Interpretation. Paleoceanography and Paleoclimatology. 2019 Feb;34(2):217–36.

33.

Koppes M, Conway H, Rasmussen LA, Chernos M. Deriving mass balance and calving variations from reanalysis data and sparse observations, Glaciar San Rafael, northern Patagonia, 1950–2005. The Cryosphere. 2011 Sep 29;5(3):791–808.

34.

Llopart M, Simões Reboita M, Porfírio da Rocha R. Assessment of multi-model climate projections of water resources over South America CORDEX domain. Clim Dyn. 2020 Jan;54(1–2):99–116.

35.

López S, López-Sandoval MF, Jung JK. New Insights on Land Use, Land Cover, and Climate Change in Human–Environment Dynamics of the Equatorial Andes. Annals of the American Association of Geographers. 2021 Jun 7;111(4):1110–36.

36.

López S, Wright C, Costanza P. Environmental change in the equatorial Andes: Linking climate, land use, and land cover transformations. Remote Sensing Applications: Society and Environment. 2017 Nov;8:291–303.

37.

Marín JC, Barrett BS, Pozo D. The tornadoes of 30–31 May 2019 in south-Central Chile: Sensitivity to topography and SST. Atmospheric Research. 2021 Feb;249:105301.

38.

Maussion F, Gurgiser W, Großhauser M, Kaser G, Marzeion B. ENSO influence on surface energy and mass balance at Shallap Glacier, Cordillera Blanca, Peru. The Cryosphere. 2015 Aug 21;9(4):1663–83.

39.

Möller M, Schneider C. Calibration of glacier volume–area relations from surface extent fluctuations and application to future glacier change. J Glaciol. 2010;56(195):33–40.

40.

Mora DE, Campozano L, Cisneros F, Wyseure G, Willems P. Climate changes of hydrometeorological and hydrological extremes in the Paute basin, Ecuadorean Andes. Hydrol Earth Syst Sci. 2014 Feb 19;18(2):631–48.

41.

Moya-Álvarez AS, Estevan R, Kumar S, Flores Rojas JL, Ticse JJ, Martínez-Castro D, et al. Influence of PBL parameterization schemes in WRF_ARW model on short - range precipitation’s forecasts in the complex orography of Peruvian Central Andes. Atmospheric Research. 2020;233.

42.

Moya-álvarez AS, Gálvez J, Holguín A, Estevan R, Kumar S, Villalobos E, et al. Extreme rainfall forecast with the WRF-ARW model in the Central Andes of Peru. Atmosphere. 2018;9(9).

43.

Moya-Álvarez AS, Martínez-Castro D, Kumar S, Estevan R, Silva Y. Response of the WRF model to different resolutions in the rainfall forecast over the complex Peruvian orography. Theoretical and Applied Climatology. 2019;137(3–4):2993–3007.

44.

Moya-Alvarez AS, Martinez-Castro D, Flores JL, Silva Y. Sensitivity Study on the Influence of Parameterization Schemes in WRF_ARW Model on Short- and Medium-Range Precipitation Forecasts in the Central Andes of Peru. Vol. 2018, ADVANCES IN METEOROLOGY. ADAM HOUSE, 3RD FLR, 1 FITZROY SQ, LONDON, W1T 5HF, ENGLAND: HINDAWI LTD; 2018.

45.

Moya-Alvarez AS, Martinez-Castro D, Kumar S, Estevan R, Silva Y. Response of the WRF model to different resolutions in the rainfall forecast over the complex Peruvian orography. Vol. 137, THEORETICAL AND APPLIED CLIMATOLOGY. SACHSENPLATZ 4-6, PO BOX 89, A-1201 WIEN, AUSTRIA: SPRINGER WIEN; 2019. p. 2993–3007.

46.

Navarro-Barboza H, Moya-Álvarez A, Luna A, Fashé-Raymundo O. Influence evaluation of PM<inf>10</inf> produced by the burning of biomass in Peru on AOD, using the WRF-Chem. Atmosfera. 2020;33(1):71–86.

47.

Nicholson L, Marín J, Lopez D, Rabatel A, Bown F, Rivera A. Glacier inventory of the upper Huasco valley, Norte Chico, Chile: glacier characteristics, glacier change and comparison with central Chile. Ann Glaciol. 2009;50(53):111–8.

48.

Ochoa A, Campozano L, Sánchez E, Gualán R, Samaniego E. Evaluation of downscaled estimates of monthly temperature and precipitation for a Southern Ecuador case study. International Journal of Climatology. 2016 Mar;36(3):1244–55.

49.

Olsson T, Kämäräinen M, Santos D, Seitola T, Tuomenvirta H, Haavisto R, et al. Downscaling climate projections for the Peruvian coastal Chancay-Huaral Basin to support river discharge modeling with WEAP. Journal of Hydrology: Regional Studies. 2017 Oct;13:26–42.

50.

Pesquero JF, Chou SC, Nobre CA, Marengo JA. Climate downscaling over South America for 1961–1970 using the Eta Model. Theor Appl Climatol. 2010 Jan;99(1–2):75–93.

51.

Pineda LE, Willems P. Multisite Downscaling of Seasonal Predictions to Daily Rainfall Characteristics over Pacific–Andean River Basins in Ecuador and Peru Using a Nonhomogeneous Hidden Markov Model. Journal of Hydrometeorology. 2016 Feb 1;17(2):481–98.

52.

Posada-Marin JA, Rendon AM, Salazar JF, Mejia JF, Camilo Villegas J. WRF downscaling improves ERA-Interim representation of precipitation around a tropical Andean valley during El Nino: implications for GCM-scale simulation of precipitation over complex terrain. CLIMATE DYNAMICS. 2019 Mar;52(5–6):3609–29.

53.

Posadas A, Duffaut Espinosa LA, Yarlequé C, Carbajal M, Heidinger H, Carvalho L, et al. Spatial random downscaling of rainfall signals in Andean heterogeneous terrain. Nonlin Processes Geophys. 2015 Jul 16;22(4):383–402.

54.

Puliafito SE, Allende DG, Mulena CG, Cremades P, Lakkis SG. Evaluation of the WRF model configuration for Zonda wind events in a complex terrain. Atmospheric Research. 2015;166:24–32.

55.

Réveillet M, MacDonell S, Gascoin S, Kinnard C, Lhermitte S, Schaffer N. Impact of forcing on sublimation simulations for a high mountain catchment in the semiarid Andes. Cryosphere. 2020;14(1):147–63.

56.

Riquetti NB, Mello CR, Beskow S, Viola MR. Rainfall erosivity in South America: Current patterns and future perspectives. Science of The Total Environment. 2020 Jul;724:138315.

57.

Rojas M. Multiply Nested Regional Climate Simulation for Southern South America: Sensitivity to Model Resolution. Monthly Weather Review. 2006 Aug 1;134(8):2208–23.

58.

Saavedra M, Junquas C, Espinoza JC, Silva Y. Impacts of topography and land use changes on the air surface temperature and precipitation over the central Peruvian Andes. Atmospheric Research. 2020;234.

59.

Santos JR, Norte F, Moreiras S, Araneo D, Simonelli S. Precipitation events prediction that causes debris flow and landslides over mountainous area on Norwest region of Mendoza Province, Argentina | Prediccion de episodios de precipitacion que ocasionan aludes en el area montañosa del noroeste de la Provincia. Geoacta (Argentina). 2015;40(1):65–75.

60.

Schaefer M, Machguth H, Falvey M, Casassa G. Modeling past and future surface mass balance of the Northern Patagonia Icefield: SURFACE MASS BALANCE OF THE NORTHERN PATAGONIA ICEFIELD. J Geophys Res Earth Surf. 2013 Jun;118(2):571–88.

61.

Schumacher V, Fernández A, Justino F, Comin A. WRF High Resolution Dynamical Downscaling of Precipitation for the Central Andes of Chile and Argentina. Frontiers in Earth Science. 2020;8.

62.

Seth A, Thibeault J, Garcia M, Valdivia C. Making Sense of Twenty-First-Century Climate Change in the Altiplano: Observed Trends and CMIP3 Projections. Annals of the Association of American Geographers. 2010 Aug 31;100(4):835–47.

63.

Shannon S, Smith R, Wiltshire A, Payne T, Huss M, Betts R, et al. Global glacier volume projections under high-end climate change scenarios. The Cryosphere. 2019 Feb 1;13(1):325–50.

64.

Shen H, Lynch B, Poulsen CJ, Yanites BJ. A modeling framework (WRF-Landlab) for simulating orogen-scale climate-erosion coupling. Vol. 146, COMPUTERS & GEOSCIENCES. THE BOULEVARD, LANGFORD LANE, KIDLINGTON, OXFORD OX5 1GB, ENGLAND: PERGAMON-ELSEVIER SCIENCE LTD; 2021.

65.

Solman SA, Sanchez E, Samuelsson P, da Rocha RP, Li L, Marengo J, et al. Evaluation of an ensemble of regional climate model simulations over South America driven by the ERA-Interim reanalysis: model performance and uncertainties. Clim Dyn. 2013 Sep;41(5–6):1139–57.

66.

Souvignet M, Gaese H, Ribbe L, Kretschmer N, Oyarzún R. Statistical downscaling of precipitation and temperature in north‐central Chile: an assessment of possible climate change impacts in an arid Andean watershed. Hydrological Sciences Journal. 2010 Mar 10;55(1):41–57.

67.

Sun X, Barros AP. Impact of Amazonian evapotranspiration on moisture transport and convection along the eastern flanks of the tropical Andes. Quarterly Journal of the Royal Meteorological Society. 2015;141(693):3325–43.

68.

Trachte K. Atmospheric moisture pathways to the highlands of the tropical Andes: Analyzing the effects of spectral nudging on different driving fields for regional climate modeling. Atmosphere. 2018;9(11).

69.

Urrutia R, Vuille M. Climate change projections for the tropical Andes using a regional climate model: Temperature and precipitation simulations for the end of the 21st century. J Geophys Res. 2009 Jan 23;114(D2):D02108.

70.

Weidemann S, Sauter T, Schneider L, Schneider C. Impact of two conceptual precipitation downscaling schemes on mass-balance modeling of Gran Campo Nevado ice cap, Patagonia. J Glaciol. 2013;59(218):1106–16.

71.

Wongchuig SC, Mello CR, Chou SC. Projections of the impacts of climate change on the water deficit and on the precipitation erosive indexes in Mantaro River Basin, Peru. J Mt Sci. 2018 Feb;15(2):264–79.

72.

Yáñez-Morroni G, Gironás J, Caneo M, Delgado R, Garreaud R. Using the Weather Research and Forecasting (WRF) Model for Precipitation Forecasting in an Andean Region with Complex Topography. Atmosphere. 2018 Aug 2;9(8):304.

73.

Zhiña D, Montenegro M, Montalván L, Mendoza D, Contreras J, Campozano L, et al. Climate Change Influences of Temporal and Spatial Drought Variation in the Andean High Mountain Basin. Atmosphere. 2019 Sep 18;10(9):558.
