# Supplementary material for: Downscaling precipitation and temperature in the Andes: applied methods and performance—a systematic review protocol
Source: Environ Evid. 2023 Dec 12;12:29. doi: 10.1186/s13750-023-00323-0 (PMC11378818; doi:10.1186/s13750-023-00323-0)
Supplement: Supplementary file 6 — Additional file 6. Meta-analysis strategy. [file 13750_2023_323_MOESM6_ESM.docx]

**README**

This file describes in a simple way the steps to perform the meta-analysis and the strategy to calculate the effect size based on the performance metrics of the downscaling methods.

**Meta analysis strategy and effect size calculation based on the performance metrics of the downscaling methods.**

- All the performance metrics reported in the research article (the ones available from Table 1) are extracted and converted to the indicated units (when necessary). An example of the data extraction sheet is in Additional File 4.
- The appropriate metrics (Table 1) will be requested or calculated by the reviewers where no performance metrics are reported, but the results are freely available as supplementary material or online.
- Once the data is extracted for all the studies, we will count the number of applications with each performance metric and select the three most frequently reported. Only those metrics will go through the next steps.

1. Is the study performing a comparison between the original data without downscaling (reanalysis, climate model outputs, satellite data) against a version of the data after downscaling (go to 2, otherwise it is not possible to include the study in the meta-analysis)
2. Calculate the log-response ratio between the performance metrics of the data without downscaling as control and the downscaled results as intervention.
3. Fit the multilevel meta-analytic model for each effect size in the software R (3 different models).
4. Calculate the heterogeneity between studies with the I-square statistic test.
5. Plot as forest and orchard plots the effect sizes of the performance metrics against the categorical moderators (sources of heterogeneity), distinguishing the downscaling method (intervention) with a particular color or marker. Each of the three selected performance metrics will have its own plot.
6. Estimate the coefficients of the meta-regressions, by including quantitative sources of heterogeneity.
